# *Pseudomonas aeruginosa* colonization enhances ventilator-associated pneumonia-induced lung injury

**DOI:** 10.1186/s12931-016-0417-5

**Published:** 2016-08-09

**Authors:** Tzyy-Bin Tsay, Yu-Zhen Jiang, Ching-Mei Hsu, Lee-Wei Chen

**Affiliations:** 1 Department of Surgery, Kaohsiung Armed Forces General Hospital Zuoying Branch, Kaohsiung, Taiwan; 2 Department of Biological Sciences, National Sun Yat-Sen University, Kaohsiung, Taiwan; 3 Department of Surgery, Kaohsiung Veterans General Hospital, 386, Ta-Chung 1st Road, Kaohsiung, Taiwan; 4 Institute of Emergency and Critical Care Medicine, National Yang-Ming University, Taipei, Taiwan

**Keywords:** Mechanical ventilation, TNF-α, IL-6, IL-1β, Neutrophil

## Abstract

**Background:**

*Pseudomonas aeruginosa* (PA) is the single-most common pathogen of ventilator-associated pneumonia (VAP). Large quantities of PA in the trachea of ventilated patients are associated with an increased risk of death. However, the role of PA colonization in PA VAP*-induced lung injury remains elusive.* This study examined the effect and mechanism of PA colonization in VAP-induced lung injury.

**Methods:**

C57BL/6 wild-type (WT) and c-Jun N-terminal kinase knockout (JNK1^−/−^) mice received mechanical ventilation for 3 h at 2 days after receiving nasal instillation of PA (1 × 10^6^ colony forming unit) or normal saline.

**Results:**

Intranasal instillation of PA or mechanical ventilation induced the expression of interleukin-6 (IL-6) in the lungs. Phospho-JNK protein expression in the lungs was significantly increased in mice receiving mechanical ventilation after PA instillation as compared with those receiving ventilation alone. Mechanical ventilation after PA instillation significantly increased the expression of tumor necrosis factor-α (TNF-α), IL-1β, and macrophage inflammatory protein-2 (MIP-2) proteins; neutrophil sequestration; and TNF-α, IL-1β, and IL-6 levels in the lungs of WT mice, but not in JNK1^−/−^ mice.

**Conclusion:**

PA colonization plays an important role in PA VAP*-induced lung injury through the induction of* JNK1-mediated inflammation. PA-induced VAP causes lung injury through JNK signaling pathway in the lungs. JNK inhibition in ICU patients with higher percentages of PA colonization may reduce VAP-induced lung injury and mortality.

## Background

Ventilator-associated pneumonia (VAP) continues to be a serious complication in patients receiving mechanical ventilation for > 48 h in the intensive care unit (ICU) [1]. VAP caused by *Pseudomonas aeruginosa* (PA) has been associated with higher case fatality rates than that by other bacteria [2, 3]. More importantly, PA is the most common multidrug-resistant pathogen that rarely causes pneumonia outside of the ICU but is responsible for a high proportion of these infections in hospitalized patients [4]. Tracheobronchial colonization is one of the most important factors for VAP and the predominant organisms responsible for infection are *Staphylococcus aureus*, PA, and *Enterobacteriaceae*. Large quantities of PA in the trachea of ventilated patients are associated with an increased risk of death [5]. However, the role of routine endotracheal aspirates (ETA) surveillance is controversial, because some studies identified the same pathogens in ETAs and VAP cultures [6, 7], whereas others found poor correlations [8]. A recent study reported that higher percentages of PA-colonized patients subsequently developed PA-induced VAP [9]. Thus, the role of PA *colonization in PA* VAP*-induced lung injury remains to be clearly defined.*

Cytokines are small proteins that communicate via intercellular signaling and can be regarded as immunomodulators for immune and inflammatory responses [10]. Human studies suggest that the release of cytokines/chemokines and the recruitment of leukocytes causes ventilator-associated lung injury [11]. Experimental models have demonstrated increased vascular permeability, higher cell count and protein concentration in the bronchoalveolar lavage fluid (BALF), and increased inflammatory cell infiltration into lung tissues in ventilator-induced lung injury (VILI) [11–15]. Interleukin-6 (IL-6), macrophage inflammatory protein (MIP-2) and tumor necrosis factor-α (TNF-α) are all involved in inflammation [16, 17]. MIP-2 is a potent leukocyte chemoattractant and plays a very important role in the pathogenesis of VILI [11]. Phosphorylated JNKs activate the oncoprotein c-Jun, which is known to form the activation protein-1 (AP-1) transcription factor as a homo- or heterodimer [18]. In mammalian cells, kinases of the JNK group are primarily activated by proinflammatory cytokines (IL-1β and TNF-α) and stress stimuli (UV radiation, pH changes, heat shock, as well as genotoxic and oxidative stress) [19]. JNKs are particularly relevant to TNF-α-mediated induction of AP-1 activity [20, 21].

Airway epithelial cells are the front-line defenders of the lungs against invading microbes by providing a physical barrier and antimicrobial activity [22]. The airway epithelial cells increase the production of mediators such as cytokines, chemokines and antimicrobial peptides to respond to such exposure [23]. In response to pathogens, the endothelial cells promote inflammation by expressing different combinations of adhesion molecules for leukocytes such as E-selectin, intercellular adhesion molecule-1 (ICAM-1) and vascular cell adhesion molecule-1 (VCAM-1) in distinct temporal, spatial and anatomical patterns [24]. Therefore, in this study, the nasal instillation of PA before mechanical ventilation in mice was used as a model to study the mechanism of PA VAP-induced lung injury. The primary objective of this study was to determine the relationship between PA colonization and VAP-induced lung injury. The secondary objective was to examine the molecular mechanisms and involvement of JNK signaling pathways in PA VAP-induced lung injury. Our results suggest that PA stimulates AMs to release mediators that activate JNK in the lungs and enhance mechanical ventilation-induced lung injury.

## Methods

### Animals

C57BL/6 (wild-type, WT) mice weighing between 18 g and 25 g were purchased from the National Laboratory Breeding and Research Center (NLBRC, Taipei, Taiwan). JNK1^−/−^ (c-Jun N-terminal kinase knockout) mice generated from the same background were transferred from Dr. Karin’s laboratory (University of California, San Diego, CA, USA). All animal procedures were in compliance with the regulations on animals used for experimental and other scientific purposes approved by the National Sun Yat-Sen University Animal Experiments Committee.

### VAP-induced lung injury

An animal model of PA VAP-induced lung injury was established. WT mice were anesthetized and instilled with 10 μl of normal saline as the control or with an equal volume of PA (ATCC 27853, 10^6^ CFU) via the nostrils into the lungs. After 2 days of PA instillation, the mice received mechanical ventilation for 3 h. The mice were sacrificed and the lungs were harvested and assayed for the expression of proinflammatory cytokines, AP-1 DNA-binding activity, and histological study. BALF was also collected for cell counting and protein concentration assay.

### Mechanical ventilation treatment

At 2 days after instillation, the mice were sacrificed or received mechanical ventilation for 3 h. Mice were anesthetized with Avertin (15 mg/kg, Sigma), and the neck was cut at 1 cm below the mouth. The muscles were separated and the trachea was opened and cannulated with a 0.5 cm 21G needle connected to a mechanical ventilator (SAR-830/P, CWE Inc., Ardmore, PA, USA) with an analog pressure output signal for 3 h. Mice were administered avertin every 20 min during the period of ventilation. The ventilation was with high stretch (tidal volume, Vt = 30 ml/kg) and without positive end expiratory pressure (PEEP).

### Tissue preparation

Mice were sacrificed and the lungs and heart were harvested. Saline (5 ml) was injected into the right ventricle using a syringe to clear the blood in the pulmonary vasculature. The lung tissue was blotted dry of surface blood and immediately stored at −80 °C for analysis.

### Preparation of BALF

For whole lung lavage, the lavage was washed with two separate injections of 0.5 ml sterile saline through a 21G needle that was cannulated 0.5 cm into the trachea. The collected BALF was used for cell counting with a hemocytometer. BALF was also centrifuged at 350 × *g* for 5 min, and the supernatants were collected and stored at −80 °C.

### Western immunoblotting

The harvested lung tissue was weighed and homogenized in protein extraction buffer (Sigma) containing proteinase inhibitor cocktail (Roche), 1 mM NaF and 1 mM Na_3_VO_4_. The homogenized samples were subjected to SDS-PAGE at 50 to 100 V for 2 h. The proteins were transferred onto the nitrocellulose membrane. The membrane was blocked with 5 % non-fat milk in TBST buffer (10 mM Tris-HCl, pH 7.5, 150 mM NaCl and 1.2 % Tween 20) at room temperature for 1 h and incubated with antibodies against TNF-α, IL-1β, IL-6, MIP-2, JNK, and phospho-JNK at room temperature for 1 h. After immunoblotting with the specific primary antibodies, the membranes were washed 3 times with TBST buffer and incubated with the secondary antibodies at room temperature for 1 h. The membranes were washed 6 to 8 times with TBST buffer and the protein bands were detected by enhanced chemiluminescence (ECL) detection reagent (Millipore).

### Enzyme-linked immunosorbent assay (ELISA)

Lung tissues were collected for TNF-α, IL-1β and IL-6 assay by using the mouse ELISA kit (eBioscience). Lung tissue was homogenized in lysis buffer (30 mM Tris, pH 7.5, 300 mM NaCl, 2 mM MgCl_2_, 10 % Triton X-100, 2 mM CaCl_2_, and 20 μg/ml of protease inhibitors) and centrifuged at 1,000 × g, 4 °C for 15 min. The supernatants was collected and used for assay. The ELISA plates were coated with capture antibodies (100 μl per well) at 4 °C for overnight. The plates were washed several times and blocked with assay buffer (200 μl per well) at room temperature for 1 h. The samples and standards were added to the plates and incubated at 4 °C for overnight. On the next day, the plates were washed several times, detection antibodies (100 μl per well) were added for 1 h and avidin-HRP (100 μl per well) was added for 30 min at room temperature. Finally, substrate 3,3′,5,5′-tetramethylbenzidine was added and incubated at room temperature for 15 min. The reaction was stopped by adding 2 N H_2_SO_4_ and the absorbance at 450 nm was measured by using an ELISA reader.

### Neutrophil infiltration in the lungs

Lung myeloperoxidase (MPO) activity has been used as a marker of lung neutrophil infiltration [25]. Lung tissues were weighed and homogenized in 50 mM potassium phosphate buffer (pH 6.0) with 0.5 % hexadecyltrimethyl- ammonium bromide. Homogenates were centrifuged at 9,500 × g, 4 °C for 10 min. An aliquot (60 μl) of supernatants was added to 939 μl of potassium phosphate buffer with 16.7 mg/ml of O-dianisidine and 0.5 % hydrogen peroxide. The rate of change in absorbance at 460 nm was measured over 2 min. One unit of MPO activity is defined as the amount of enzyme that reduces 1 μmole of peroxide per min and the data were expressed as units per gram of lung tissue (Units/g tissue).

### Histological study

Tissue samples were collected and fixed in 4 % formalin for 24 h. The samples were embedded in paraffin, cut into 3-5 μm sections, and stained with hematoxylin and eosin. Pulmonary edema and infiltration of inflammatory cells were observed.

### Statistics

All data are analyzed by one-way analysis of variance or *T*-test analysis of variance (ANOVA), followed by Turkey’s Multiple Comparison Test. All values in the figures and text are expressed as mean ± standard error of the mean. *P* values of less than 0.05 are considered to be statistically significant.

## Results

### Mechanical ventilation after PA instillation induces lung injury

**Fig. 1:**
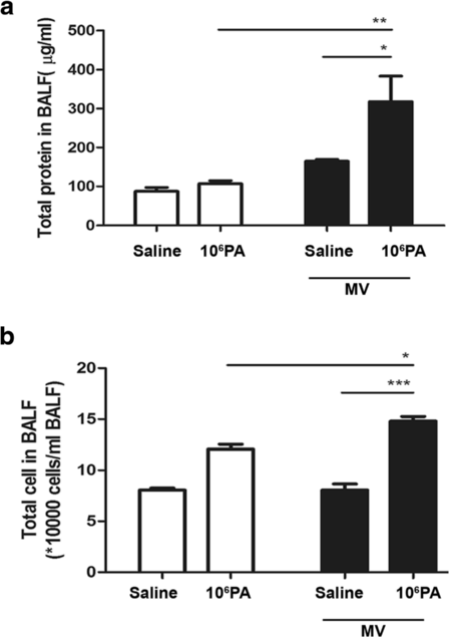
Effects of *Pseudomonas aeruginosa* (PA) colonization on mechanical ventilation-induced lung injury. WT mice were intranasally instilled with live PA (1 × 10^6^ CFU) or normal saline at 2 days before receiving ventilation for 3 h, and lung tissues were harvested and assayed. PA instillation (1 × 10^6^ CFU) before mechanical ventilation increased the total number of cells (**a**) and protein concentration (**b**) in BALF of WT mice. PA, *Pseudomonas aeruginosa*; CFU, colony forming unit; BALF, bronchoalveolar lavage fluid; MV, mechanical ventilation for 3 h. *P < 0.05, **P < 0.01, ***P < 0.001. *n* = 5-6/group

The total number of cells and protein concentration in BALF were increased (1.5 to 2-fold) in mice receiving mechanical ventilation after 10^6^ PA instillation as compared with those receiving ventilation or PA instillation alone (Fig. 1a, b). These results suggest that mechanical ventilation after PA instillation enhances PA instillation-induced acute lung injury.

### Mechanical ventilation after PA instillation induces proinflammatory cytokine expression in the lungs

**Fig. 2:**
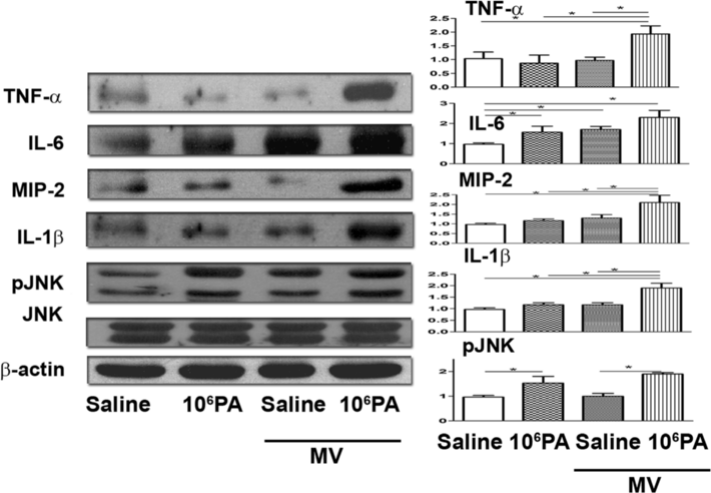
Mechanical ventilation after PA instillation induced cytokine as well as phospho-JNK expression in the lungs. Protein expression of proinflammatory cytokines (TNF-α, IL-1β, MIP-2, and IL-6), c-Jun NH2-terminal kinase (JNK), and phospho-JNK in the lungs of mice after different treatments was examined by Western blotting. CFU, colony forming unit; PA, *Pseudomonas aeruginosa*; MV, mechanical ventilation for 3 h. *P < 0.05, **P < 0.01. *n* = 3/group

To study the role of proinflammatory cytokines/chemokines in PA VAP-induced lung injury, proteins and mRNAs in the lungs were analyzed. PA (10^6^ CFU) instillation significantly increased the expression of IL-6 protein in the lungs as compared with saline injection group (Fig. 2a). Mechanical ventilation also increased the expression of IL-6 protein in the lungs. The expression of TNF-α, IL-1β, IL-6, and MIP-2 protein in the lungs was significantly increased in mice receiving mechanical ventilation after PA (10^6^ CFU) instillation as compared with those receiving PA or ventilation alone (Fig. 2). Phospho-JNK protein expression in the lungs was significantly increased in mice receiving mechanical ventilation after PA (10^6^ CFU) instillation as compared with those receiving ventilation alone (Fig. 2).

### Mechanical ventilation after PA instillation induces cytokine production in the lungs

**Fig. 3:**
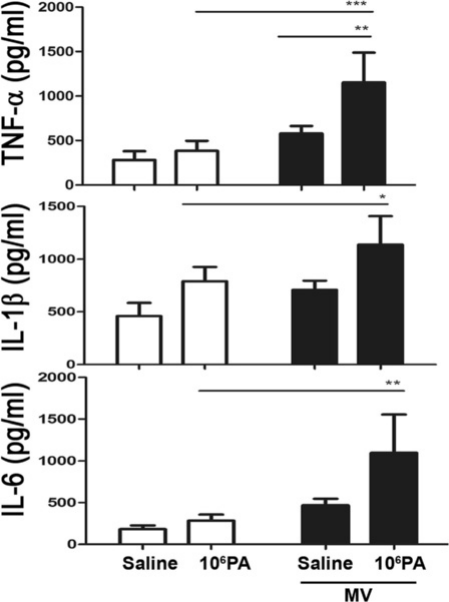
Mechanical ventilation after PA instillation induced the production of TNF-α, IL-1β and IL-6 in the lungs of WT mice. PA (1 × 10^6^ CFU) intranasal instillation and mechanical ventilation increased TNF-α, IL-1β, and IL-6 levels in the lungs as compared to those in the PA instillation or mechanical ventilation alone group

TNF-α, IL-1β and IL-6 levels in the lungs were determined by enzyme-linked immunosorbent assay (ELISA). Mechanical ventilation after PA instillation in mice induced a significant increase in the TNF-α levels in the lungs as compared with those receiving ventilation or PA instillation alone (Fig. 3). Mechanical ventilation after PA instillation in mice induced a significant increase in the IL-1β and IL-6 levels in the lungs as compared with those receiving PA instillation alone (Fig. 3).

### Mechanical ventilation after PA instillation-induced proinflammatory cytokine expression in the lungs after mechanical ventilation is prevented in JNK1^−/−^ mice

**Fig. 4:**
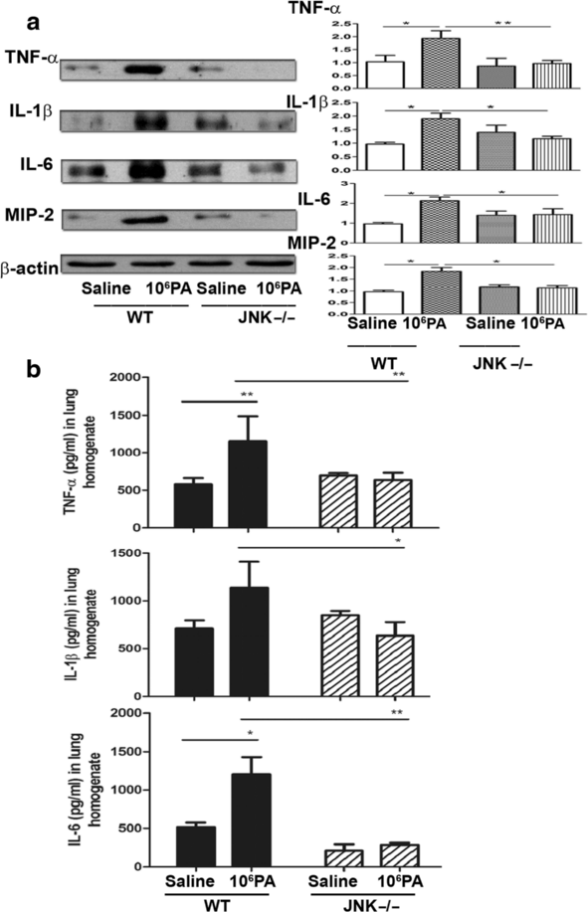
Mechanical ventilation after PA instillation induced protein expression of cytokines in the lungs of WT mice, but not in JNK1^−/−^ mice. **a** The protein expression of proinflammatory cytokines (TNF-α, IL-1β, MIP-2, and IL-6) in the lungs of WT and JNK1^−/−^ mice after different treatment was examined by Western blotting. *n* = 3/group. **b** The levels of proinflammatory cytokines (TNF-α, IL-1β and IL-6) in the lungs of WT and JNK1^−/−^ mice after saline or 1 × 10^6^ CFU PA instillation and mechanical ventilation treatment. CFU, colony forming unit; PA, *Pseudomonas aeruginosa*. *P < 0.05, **P < 0.01. *n* = 4-6/group

The protein expression of cytokines in the lungs in JNK1^−/−^ mice receiving mechanical ventilation after PA instillation was determined to examine the role of JNK in PA VAP-induced lung injury. Mechanical ventilation after PA instillation induced a significant increase in the expression of TNF-α, IL-1β, IL-6 and MIP-2 proteins (Fig. 4a) in the lungs of WT mice but not in JNK1^−/−^ mice. Mechanical ventilation after PA instillation induced TNF-α, IL-1β and IL-6 in the lungs of WT mice but not in JNK1^−/−^ mice (Fig. 4b). These results suggest that JNK signaling pathway is crucial in mechanical ventilation after PA instillation-induced production of proinflammatory cytokines in the lungs.

### Mechanical ventilation after PA instillation induces MPO activity in the lungs of WT mice but not in JNK1^−/−^ mice

**Fig. 5:**
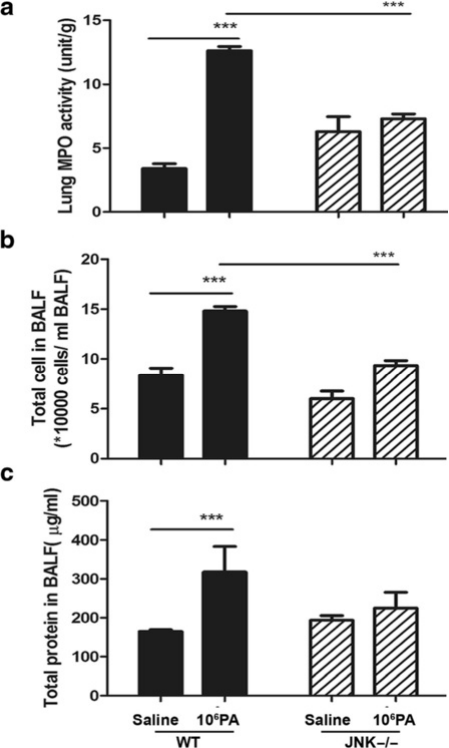
Effects of mechanical ventilation after PA or normal saline instillation on lung MPO activity, total number of cells, and total protein concentration in BALF of WT and JNK1^−/−^ mice. **a** Mechanical ventilation after saline or 1 × 10^6^ CFU PA instillation induced lung MPO activity in WT mice but not in JNK1^−/−^ mice. *n* = 5-6/group. **b** Mechanical ventilation after PA instillation increased the total number of cells in BALF in WT mice but not in JNK1^-/-^ mice. **c** Mechanical ventilation after PA instillation increased the total protein concentration in BALF of WT mice but not in JNK1^−/−^ mice. *n* = 5-6/group. PA, *Pseudomonas aeruginosa.* *P < 0.05, **P < 0.01, ***P < 0.001

To determine the role of JNK activation in mechanical ventilation after PA instillation-induced lung injury, the pulmonary MPO activity and protein concentration in BALF in JNK1^−/−^ mice were examined. JNK1^−/−^ mice receiving mechanical ventilation after PA instillation showed a significant decrease in the pulmonary MPO activity as compared to that in the WT mice. Moreover, there was no significant difference in the MPO activity between mechanical ventilation after PA instillation and that after saline instillation in JNK1^−/−^ mice (Fig. 5a), suggesting that PA instillation had no effect on ventilation-induced lung injury in JNK1^−/−^ mice. This finding indicates that PA colonization enhances mechanical ventilation-induced lung injury through JNK signaling pathway.

### PA colonization enhances mechanical ventilation-induced total number of cells and protein concentration in BALF of WT mice but not in JNK1^−/−^ mice

To determine the role of JNK activation in mechanical ventilation after PA instillation-induced lung injury, the total number of cells and protein concentration in BALF of JNK1^−/−^ mice were examined. Mechanical ventilation after PA instillation induced a significant increase in the total number of cells as well as protein concentration in BALF of WT mice but not in JNK1^−/−^ mice as compared with the ventilation alone group (Fig. 5b, c). This observation indicates that PA colonization enhances mechanical ventilation-induced lung injury through JNK signaling pathway.

### Mechanical ventilation after PA instillation induces neutrophils in BALF in WT mice as compared with JNK1^−/−^ mice

**Fig. 6:**
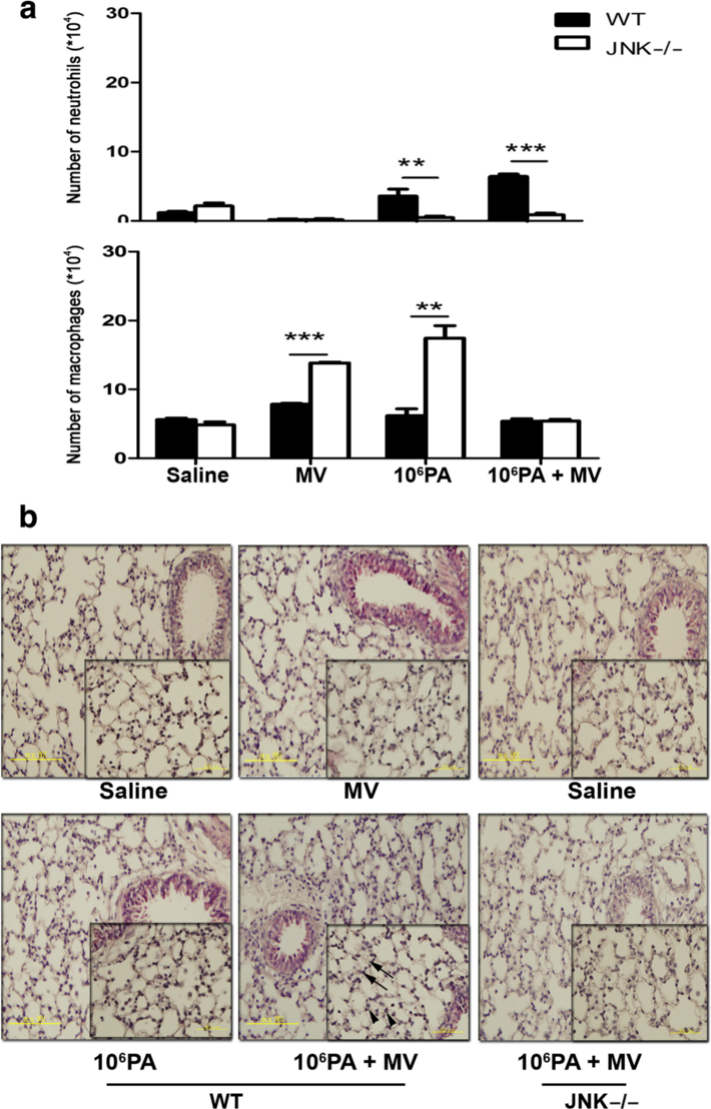
Effects of mechanical ventilation after PA or normal saline instillation on number of neutrophils and macrophages in BALF of WT and JNK1^−/−^ mice. **a** Mechanical ventilation after saline or 1 × 10^6^ CFU PA instillation induced a significant increase of neutrophils in BALF of WT mice but not in JNK1^−/−^ mice. *n* = 3-4/group. **b** The extent of neutrophil infiltration (arrowhead) and alveolus swelling (arrow) in the lungs were observed by hematoxylin and eosin staining. Mechanical ventilation after PA instillation induced significant parenchyma swelling and cell infiltration in WT mice as compared with JNK1^−/−^ mice. MV, mechanical ventilation for 3 h; PA, *Pseudomonas aeruginosa*. **P < 0.01, ***P < 0.001

The levels of neutrophils and macrophages in BALF collected from both WT and JNK1^−/−^ mice were determined using cytospin techniques. Mechanical ventilation after PA instillation induced a significant increase of neutrophils in BALF in WT mice as compared with those in JNK1^−/−^ mice (Fig. 6a). The effects of mechanical ventilation after PA instillation-induced lung injury were also evaluated by histological examination of the lungs. The extent of swelling of the parenchyma and alveoli and cell infiltration were more significant in mice receiving mechanical ventilation after nasal instillation of 10^6^ CFU of PA as compared with mice receiving ventilation alone (Fig. 6b). Mechanical ventilation after PA instillation induced significant parenchyma swelling and cell infiltration in WT mice as compared with JNK1^−/−^ mice (Fig. 6b).

## Discussion

Large quantities of PA in the trachea are associated with an increased risk of death in mechanically ventilated patients [5]. The effect and mechanism of PA colonization on PA VAP*-induced lung injury has not been clearly defined.* Ranieri et al. previously demonstrated that in patients using controlled mechanical ventilation with a respiratory rate of 10–15/min and a tidal volume targeted to maintain the PaCO2 between 35 and 40 mmHg, a significant increase of IL-1β, IL-6, and IL-1 receptor agonist in BALF was observed at 24 h after ventilation [26]. In our study, mechanical ventilation for 3 h induced IL-6 protein expression and a mild increase of TNF-α, IL-1β, and IL-6 levels in the lungs. However, PA colonization after mechanical ventilation significantly increased TNF-α, IL-1β, and IL-6 levels in the lungs as compared with the PA instillation alone group. Moreover, PA colonization significantly increased the phosphor-JNK expression in the lungs after mechanical ventilation as compared with the PA instillation alone group. The effects of enhancement of PA colonization on mechanical ventilation-induced TNF-α, IL-1β, and IL-6 levels in the lungs and the total number of cells as well as protein concentration in BALF were prevented in JNK1^−/−^ mice. Taken together, our data suggest that PA colonization plays an important role in PA VAP*-induced lung injury and that the mechanism is through the induction of* JNK1-mediated inflammatory reaction in the lungs.

PA is the single-most common pathogen that accounted for 26 % of VAP patients (4/1000 ventilator days) [27]. In this study, an animal model of PA VAP was established. Neutrophil infiltration not only plays an important role in inflammation but is also a major cause of tissue damage. WT mice instilled with PA (PA colonization) had more neutrophil infiltration than those instilled with normal saline and neutrophil infiltration was significantly increased after ventilation with PA colonization. These results suggest that mechanical ventilation after PA instillation induces lung injury through the enhancement of PA -induced inflammatory process in the lungs. The total number of cells and total protein concentration in BALF were increased in mice receiving mechanical ventilation after PA instillation. Histological studies further suggest that mechanical ventilation after PA instillation induces neutrophil infiltration, and swelling of the parenchyma and alveolus. The expression of the proinflammatory cytokines (TNF-α, IL-1β and IL-6) proteins was significantly increased in WT mice receiving mechanical ventilation after PA instillation. Prior instillation of PA significantly increased the levels of TNF-α, IL-1β and IL-6 in the lungs of WT mice after mechanical ventilation. These findings imply that an intense inflammatory reaction occurs in mice receiving mechanical ventilation after PA instillation in the lungs. Our data indicate that mechanical ventilation can greatly enhance PA colonization-induced inflammation and that PA colonization is closely related with PA VAP-induced lung injury.

In this study, JNK1^−/−^ mice were used to investigate the role of JNK activation in PA VAP-induced lung injury. Prior instillation with PA had no effect on mechanical ventilation-induced MPO activity in the lungs, total number of cells, and protein concentration in BALF of JNK1^−/−^ mice. In contrast, mechanical ventilation after PA instillation significantly increased the lung MPO activity, total number of cells and protein concentration in BALF of WT mice. PA instillation before ventilation did not change the protein expression of TNF-α, IL-1β, IL-6 and MIP-2 in the lungs of JNK1^−/−^ mice in comparison with an enhancement by PA instillation in WT mice. Moreover, the levels of TNF-α, IL-1β and IL-6 in the lungs were not elevated in JNK1^−/−^ mice. These results suggest that PA instillation had no effect on mechanical ventilation-induced lung injury in JNK1^−/−^ mice, which indicates that PA colonization enhances ventilation-induced lung injury through JNK signaling pathway. This further corroborates that JNK signaling pathway in the lungs is critical in PA VAP-induced lung injury.

## Conclusions

The molecular mechanisms of PA VAP-induced lung injury could be better understood by this study. PA colonization enhances mechanical ventilation-induced neutrophil infiltration and lung injury through JNK activation in the lungs. M*echanical ventilation enhances* PA colonization*-induced* TNF-α, IL-1β, and IL-6 levels in the lungs, *which increase neutrophil infiltration and lung injury.* These observations imply that JNK inhibition may be helpful to reduce the development of PA VAP-induced lung injury.
